# CXCR4 antibody treatment suppresses metastatic spread to the lung of intratibial human osteosarcoma xenografts in mice

**DOI:** 10.1007/s10585-013-9632-3

**Published:** 2014-01-04

**Authors:** Patrick Brennecke, Matthias J. E. Arlt, Carmen Campanile, Knut Husmann, Ana Gvozdenovic, Tiziana Apuzzo, Marcus Thelen, Walter Born, Bruno Fuchs

**Affiliations:** 1 Laboratory for Orthopedic Research, Department of Orthopedics, Balgrist University Hospital, 8008 Zurich, Switzerland; 2 Institute for Research in Biomedicine, 6500 Bellinzona, Switzerland

**Keywords:** CXCR4-CXCL12 axis, Lung metastases, *lacZ* transduction, Osteosarcoma mouse model

## Abstract

Current combined surgical and neo-adjuvant chemotherapy of primary metastatic osteosarcoma (OS) is ineffective, reflected by a 5-year survival rate of affected patients of less than 20 %. Studies in experimental OS metastasis models pointed to the CXCR4/CXCL12 homing axis as a novel target for OS metastasis-suppressive treatment. The present study investigated for the first time the CXCR4-blocking principle in a spontaneously metastasizing human 143B OS cell line-derived orthotopic xenograft mouse model. The highly metastatic 143B cells, unlike the parental non-metastatic HOS cells, express functional CXCR4 receptors at the cell surface, as revealed in this study by RT/PCR of gene transcripts, by FACS analysis with the monoclonal anti CXCR4 antibody 12G5 (mAb 12G5) and by CXCL12 time- and dose-dependent stimulation of AKT and ERK phosphorylation. A significantly (*p* < 0.05) higher CXCL12 dose-dependent chemotactic response of 143B compared to HOS cells in a Boyden chamber trans-well migration assay suggested a crucial role of the CXCL12/CXCR4 homing axis in 143B cell lung metastasis. Repetitive treatment of mice with 143B cell-derived intratibial tumors given intravenous bolus injections of mAb12G5 indeed inhibited significantly (*p* *<* 0.01) the number of X-gal-stainable lung micrometastases of *lacZ*-transduced 143B cells. Antibody treatment had also a mild inhibitory effect on primary tumor growth associated with remarkably less osteolysis, but it did not affect the number of developing lung macrometastases. In conclusion, these results demonstrate considerable potential of high-affinity CXCR4-blocking agents for OS tumor cell homing suppressive treatment in metastasizing OS complementary to current (neo)-adjuvant chemotherapy.

## Introduction

Osteosarcoma (OS) is the most common primary bone neoplasia in children and young adolescents [1]. It has an incidence of 3 persons per million and year and a median peak at 16 years [2]. Multi-agent chemotherapy resulted in disease-free survival of 60–70 % of the patients with localized disease [3]. These improvements made limb sparing surgery possible in 85–90 % of the cases without compromising the outcome compared to amputation [4]. Despite this progress for patients with localized disease, patients with metastases still have a very low mean 5-year survival rate of approximately 20 % [5–7]. The low survival rate of patients with metastatic disease is in part related to the fact that, until now, effective treatment targeting clinically non-detectable micro-metastases does not exist [8, 9]. Consequently, these non-treatable metastases eventually grow to life-threatening lung nodules with a fatal outcome.

Metastasis is a complex multistep process that includes local tissue invasion of metastatic cells, their survival in the circulation, “homing” to and extravasation in a secondary organ and proliferation/growth at the metastatic sites [10, 11]. Chemokines interacting with chemokine receptors expressed by metastasizing tumor cells have crucial roles in directing migrating cells towards secondary organs. This is well documented for the chemokine CXCL12 and the chemokine receptor CXCR4. Mueller and co-workers initially postulated and discovered the CXCL12–CXCR4 homing axis in cancer metastasis [12, 13]. They demonstrated that CXCR4 expressing breast cancer cells preferentially migrated towards protein extracts of the lung, which expresses CXCL12 abundantly. Chemo-attraction by lung tissue extracts was abolished by CXCL12-neutralizing antibodies and by preincubation of breast cancer cells with CXCR4-blocking antibodies. Meanwhile, CXCL12 and CXCR4 were found to be instrumental for the development and progression of numerous different cancer types of epithelial, haematopoietic or mesenchymal origin [14–17]. It is noteworthy that, in normal physiology, CXCL12 and CXCR4 are important for the development of the CNS, the heart and the immune system. Moreover, they are involved in angiogenesis and stem cell trafficking and in proliferation and apoptosis.

At the cellular level, binding of CXCL12 to CXCR4 was shown to stimulate intracellular calcium flux, to activate AKT and ERK signaling pathways, and to up-regulate the formation of focal adhesions, which ultimately results in increased migration along gradients of locally expressed and secreted chemokines [18–20].

In OS patients, overall disease-free and metastasis-free survival was found to be inversely related to the expression of CXCR4-encoding mRNA in primary tumor tissue [21]. Furthermore, a tissue microarray analysis showed that CXCR4 expression correlated significantly with the expression of VEGF and that co-expression of CXCR4/VEGF was an indicator for poor patient survival [22, 23]. Experimentally, proof of principle studies demonstrated that inhibition of CXCL12-evoked CXCR4 signaling by respective blocking agents inhibited metastasis in experimental models, achieved by intravenous tumor cell injection [24–26]. However, these models did not reproduce most of the complex processes characteristic for the metastatic cascade.

Consequently, we reinvestigated in the present study the metastasis suppressive potential of the CXCR4 blocking principle in a human 143B OS cell line-derived spontaneously metastasizing intratibial OS mouse model that closely mimics the human disease [27]. Primary intratibial tumors were provoked by injection of highly metastatic human 143B OS cells stably transduced with a constitutively expressed *lacZ* gene (143B-*lacZ* cells). This allowed post-mortem X-gal staining of primary tumor tissue and, more importantly, of lung metastases down to the single cell level as reported [28]. Tumor-bearing mice were treated at 4-day intervals by tail-vein bolus injections of the monoclonal anti-CXCR4 antibody (mAb 12G5), known to block the binding of CXCL12 to CXCR4 and to inhibit CXCL12-evoked chemotaxis in vitro [29, 30]. Tumor-bearing mice injected with control mouse IgG were considered as untreated controls. CXCR4 antibody treatment inhibited 143B-*lacZ* cell homing to the lung and had a mild inhibitory effect on primary tumor osteolysis and growth [31, 32]. Thus, the results of the present study demonstrate for the first time efficacy of the metastasis suppressive CXCR4 blocking principle in an orthotopic, spontaneously metastasizing OS model that mimics most aspects of the human disease.

## Materials and methods

### Cell lines

HOS cells were purchased from the American type culture collection (Rockville, MD) and 143B cells from the European collection of cell cultures (Salisbury, UK) [31]. Both cell lines were grown in Dulbecco’s modified Eagle medium (DMEM) (4.5 g/l glucose) and Ham F12 (F12) medium (1:1) supplemented with 2 mM l-glutamine and 10 % fetal calf serum (FCS) (GIBCO, Basel, Switzerland) (cell culture medium) in a humidified atmosphere of 95 % air and 5 % CO_2_ at 37 °C.

### RNA isolation, reverse transcription (RT), and polymerase chain reaction (PCR)

Total RNA was isolated from individual cell lines with TriReagent (Sigma-Aldrich, St Louis, MO) according to the manufacturer’s protocol. The RNA was quantified by measuring the absorption of extracts at 260 nm. The integrity of the RNA was assessed by agarose gel-electrophoresis. The expression levels of mRNA encoding CXCR4 were determined by semi-quantitative RT/PCR with *Gapdh* transcripts as a reference. cDNA was synthesized from 1 μg of total RNA with the High Capacity cDNA reverse transcription kit (Applied Biosystems Inc., Foster City, CA) and the protocol recommended by the supplier. PCR reactions of 50 μl final volume contained 1X PCR buffer, 0.4 μl of the RT reaction, 1.25 U Taq polymerase (5Prime), each dNTP at 200 μM concentration, and forward and reverse primers specific for GAPDH (Forward: 5′-TGA ACG GGA AGC TCA CTG GCA TGG -3′; Reverse: 5′-TGG GTG TCG CTG TTG AAG TCA GAG GA GA-3′) and for CXCR4 (Forward: 5′- GAG TGC TCC AGT AGC CAC CGC ATC-3′; Reverse: 5′-TCC GTC ATG CTT CTC AGT TTC TTC TGG-3′) encoding cDNA at 0.2 μM concentration. After initial denaturation at 94 °C for 3 min, cDNA was amplified by 27 (GAPDH) or 40 (CXCR4) PCR-cycles (denaturation at 94 °C for 40 s, primer pair-dependent annealing at 67 °C (GAPDH) or 69 °C (CXCR4) for 40 s, and elongation at 72 °C for 20 s) followed by final elongation at 72 °C for 7 min. PCR products were analyzed by agarose gel electrophoresis.

### FACS analysis of CXCR4 expression

HOS and 143B cells grown to 50 % confluence were detached with MACS buffer (PBS, 0.05 % ethylenediaminetetraacetic acid (EDTA) and 2 % FCS). They were then transferred to 5 ml Polystyrene FACS tubes (BD FALCON™, Franklin Lakes, NJ, USA) collected by centrifugation at 1,600 rpm and 4 °C for 5 min and resuspended. Subsequently, the cells were incubated with 5 μg/ml of CXCR4 antibody, clone 12G5 (R&D System, Abingdon, UK), at 4 °C for 30 min. The cells were then washed twice with PBS and incubated at 4 °C for 20 min with 5 μg/ml of Alexa-conjugated goat anti-mouse IgG (Invitrogen, Carlsbad, CA). The cells were again washed twice with PBS and filtered through PARTEC CELL TRICS filters (50 μm pore size, Görlitz, Germany). The percentage of cells expressing CXCR4 at the cell surface was determined with a FACS Aria/Calibur device (Beckton Dickinson, Allschwil, Switzeland).

### Analysis of CXCR4 signaling on Western Blots

Cells were grown to 80 % confluence and starved overnight in serum-free RPMI 1640 medium supplemented with 0.1 % BSA (Sigma-Aldrich, St Louis, MO). The next day, the cells were detached (PBS with 0.05 % EDTA), washed with PBS and stimulated in suspension with 100 ng/ml (12.5 nM) human recombinant CXCL12 (R&D System, Abingdon, UK) at 37 °C for indicated time periods. Incubations were stopped by the addition of ice-cold PBS. Cells were then collected by centrifugation and lysed on ice for 45 min in buffer containing NaF, orthovanadate and protease inhibitors (Roche, Basel, Switzerland). Cell lysates were cleared by centrifugation and mixed with an equal volume of 2 × Laemmli sample buffer and incubated at 95 °C for 5 min. Proteins were separated by SDS-PAGE and transferred to nitrocellulose membranes. Phospho(p)-AKT, total AKT, p-ERK and total ERK were detected by incubation at 4 °C overnight with respective primary antibodies (Cell Signalling Technology Inc., Denver, MA) followed by incubation at RT for 1 h with secondary horseradish peroxidase conjugated anti-rabbit IgG (Santa Cruz Biotechnology Inc., Heidelberg, Germany). The proteins were then visualized with Immobilon chemiluminescence substrate (Millipore, Billerica) and quantified with a VersaDoc™ Imaging System (Bio-Rad Laboratories, Munich, Germany). Antibody blocking experiments were essentially performed like the stimulation experiments described above, except that 143B and HOS cells were kept adherent and pre-incubated 30′ with either 20 μg (ml non-specific IgG (Jackson Immuno Research Rheinfeld, Switzerland) or 20 μg/ml of the highly specific 12G5 (anti-CXCR4) (R&D System, Abingdon, UK), before stimulating for 10′ with 100 ng/ml SDF-1.

### Chemotaxis assay

Chemotaxis assays were performed in triplicates in 48-well Boyden chambers (NeuroProbe, Gaithersburg, MD) with polyvinylpyrrolidone-free polycarbonate membranes of 8 μm pore size. Cells grown to 50 % confluence were harvested and resuspended in chemotaxis medium composed of DMEM and F12 medium (1:1) supplemented with 2 mM l-glutamine and 0.2 % FCS. 2×10^4^ cells in 50 μL chemotaxis medium were then added to the upper chamber of each well. Chemotaxis medium containing indicated concentrations of CXCL12 was added to the bottom chamber and chemotaxis was allowed under cell culture conditions for 4 h. Cells adhering to the upper surface of the membrane were removed with a rubber policeman. Cells that had migrated through the filter and adhered to the lower surface of the membrane were fixed and stained with Diff-Quik^®^ (Medion Diagnostics AG, Düdingen Switzerland). They were counted under the microscope in five randomly selected fields at 100-fold magnification.

### *LacZ* gene transduction of OS cell lines

Retroviral particles for stable *lacZ* gene transduction (*lacZ*-retrovirus) of OS cell lines were prepared as reported [28]. HOS and 143B cells were grown to subconfluence and infected with *lacZ*-retrovirus by incubation for 2 h with 1 ml virus-containing medium supplemented with 8 μg/ml polybrene (Sigma-Aldrich, St Louis, MO). The cells were then cultured for 7 days in medium containing 1,200 μg/ml G-418 (Invitrogen, Carlsbad, CA). Selection of *lacZ* transduced HOS (HOS-*lacZ*) and 143B (143B-*lacZ*) cells was monitored over time in aliquots of cells fixed with 4 % formaldehyde for 20 min and then stained with X-gal at 37 °C overnight.

### CXCR4 antibody treatment of SCID mice with intratibial 143B/*lacZ* cell-derived OS

Female 6–8 week old severe combined immunodeficient (SCID) were purchased from Charles River Laboratories (Sulzfeld, Germany) and maintained under OHB conditions. Animal care and experimental procedures were in accordance with institutional guidelines and approved by the Ethics Committee of the Veterinary Department, Canton of Zurich, Switzerland (License Number 169/2009). 143B-*lacZ* cells were grown to subconfluence, detached with Trypsin/PBS/0.05 % EDTA and resuspended in PBS/0.05 % EDTA. The single cell suspension contained 5x10^7^ cells/ml. Ten microliter aliquots of the cell suspension equivalent to 5x10^5^ cells were orthotopically injected into the medullar cavity of the left tibia of individual mice. After tumor cell injection, the mice were randomly divided into two groups. Treatment was initiated on the day of tumor cell injection and continued in 4-day intervals by bolus tail-vein injections of 100 μl PBS containing 200 μg mouse IgG (Jackson Immuno Research Rheinfeld, Switzerland) (control group) or 200 μg CXCR4 12G5-antibodies (treatment group). Tumor development and osteolysis were monitored by X-ray once a week. Tumor volume was calculated from caliper rule measurements of the width and the length of the tumor-bearing tibia using the following formulas: leg volume = length x (width)^2^ x 0.5; tumor volume = leg Volume on day X – leg Volume on day 0; (X = 0, 4, 12, 16 and 20 days after tumor cell injection). All mice were sacrificed on experimental day 20 and the organs were prepared as reported [30]. Lungs were further prepared as previously reported [28, 33]. *lacZ* gene expressing metastatic tumor cells were visualized by X- Gal staining as described [34]. Briefly, lung tissue was refixed with 2 % formaldehyde and 0.2 % glutaraldehyde in PBS at room temperature for 1 h, washed 3 times with PBS and then stained in X-Gal solution at 37 °C for at least 3 h.

Micrometastases, defined as indigo-blue stained foci smaller than 0.1 mm in diameter and macrometastases, defined as indigo-blue stained foci larger than 0.1 mm in diameter, on the surface of whole-mounts of lungs, were counted. Indigo-blue stained foci were counted on the surface of whole-mounts of lungs.

### Statistical analysis

Results are presented as mean ± standard error of the mean (SEM). The values of the primary tumor volume and the numbers of metastases were transformed to a logarithmic scale before they were analyzed for significance by 2-way ANOVA test with GraphPad Prism^®^ 5.01 software.

## Results

### Expression and signaling of CXCR4 in HOS and 143B cells

**Fig. 1:**
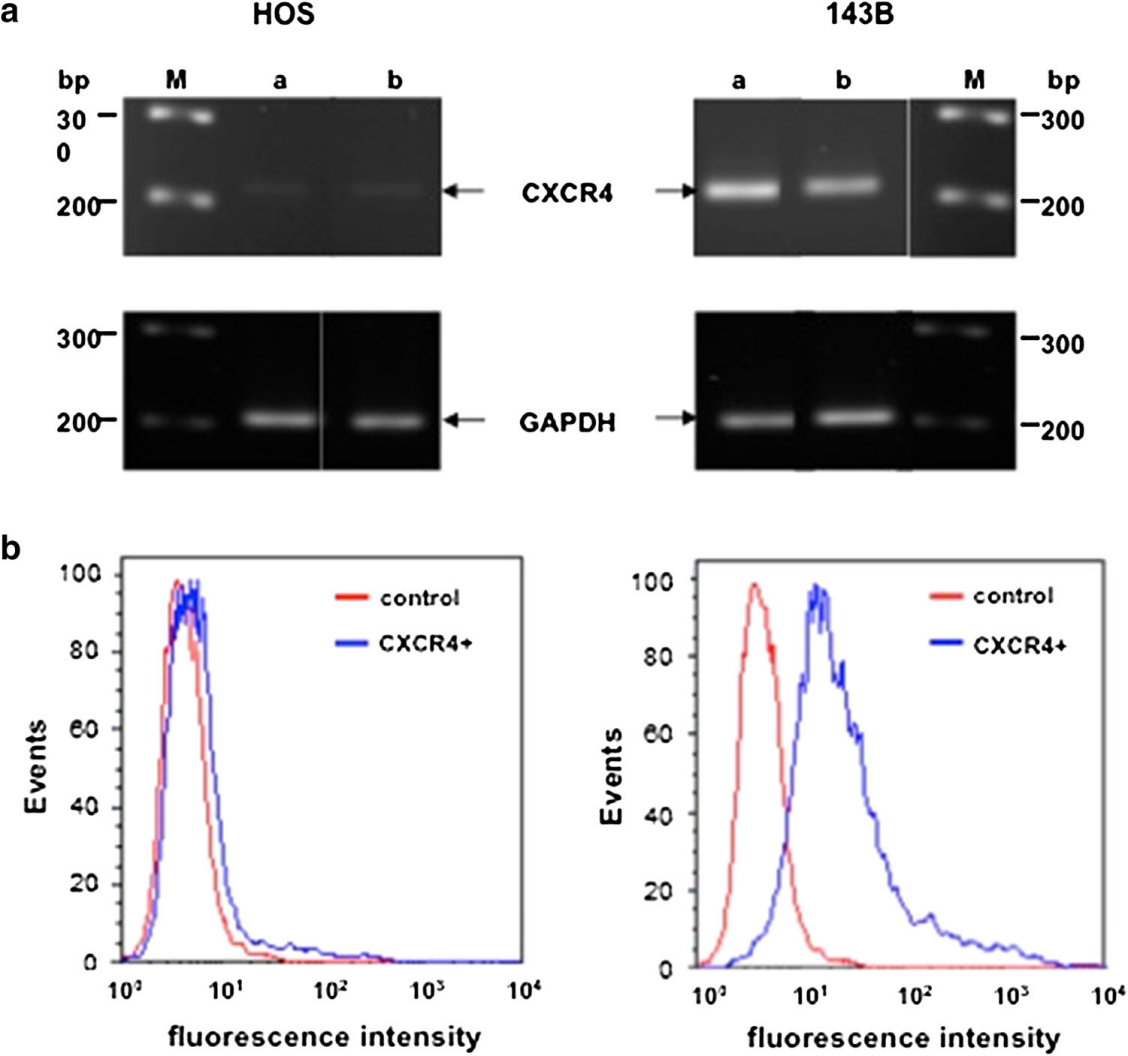
Expression of CXCR4 in the parental low metastatic human osteosarcoma HOS cell line and the highly metastatic 143B subline. **a** semi-quantitative RT/PCR analysis of *Cxcr4* and *Gapdh* (reference) transcript levels in total RNA isolated from 50 % (*a*) and 100 % (*b*) confluent cells, *M* marker lanes. **b** FACS analysis of indicated cell lines processed for cell surface immunostaining of CXCR4 in the absence (control) or presence (CXCR4+) of primary 12G5 CXCR4 antibodies

Semi-quantitative RT/PCR analysis of total RNA extracted from 50 % to 100 % confluent HOS and 143B cells detected only trace amounts of CXCR4-encoding transcripts in HOS cells compared to those observed in 143B cells (Fig. 1a). These results were in good agreement with the expression of CXCR4 on the surface of HOS and 143B cells analyzed by FACS (Fig. 1b). CXCR4 cell surface immunostaining was only recognized in 3 ± 1 % of the HOS cells analyzed, whereas 75 ± 5 % of the 143B cells expressed immune-detectable CXCR4 at the cell surface.

**Fig. 2:**
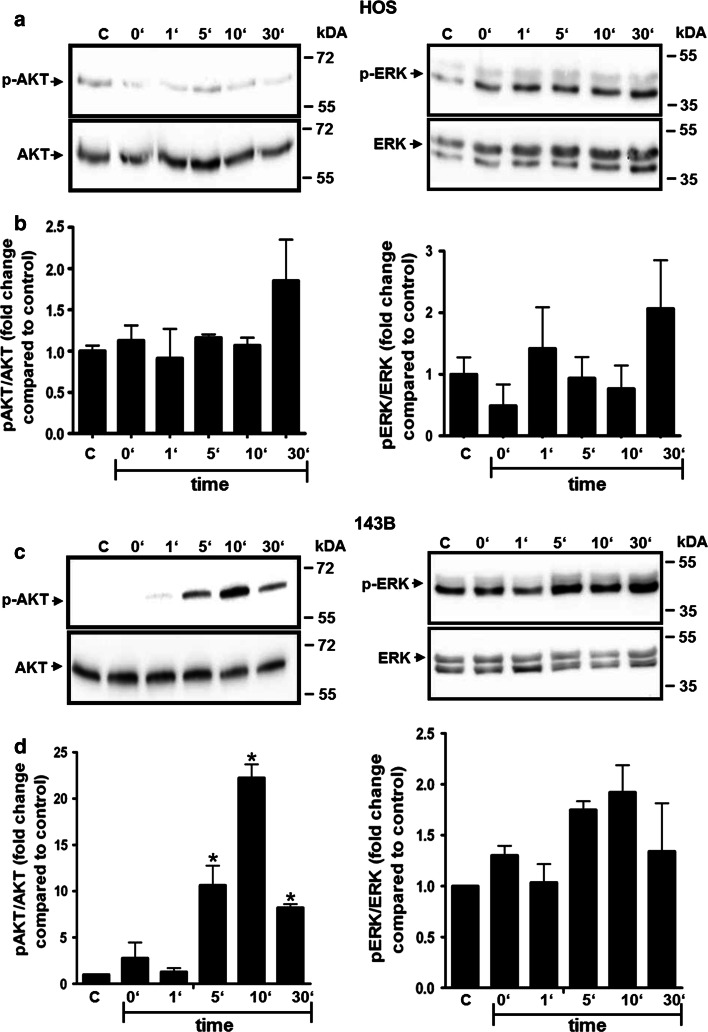
Time-dependent stimulation of AKT and ERK phosphorylation by CXCL12 in HOS and 143B cells. **a, b** western blot analysis of extracts of HOS (**a**) and of 143B (**c**) cells incubated with 12.5 nM CXCL12 for indicated time periods. The levels of p-AKT and of total AKT (*left panels*) and of p-ERK and of total ERK (*right panels*) are shown. *Data* shown in **a** and **c** are representative for at least 3 independent experiments. **b, d** Quantitative analysis of the time-dependent stimulation of AKT (*left*) and ERK (*right*) phosphorylation in HOS (*b*) and143B (*d*) cells; *Data* indicate mean ± SEM (*n* = 3) levels of p-AKT and p-ERK normalized to total AKT and total ERK, respectively, at indicated time points; *p ≤ 0.05 compared to control (C)

**Fig. 3:**
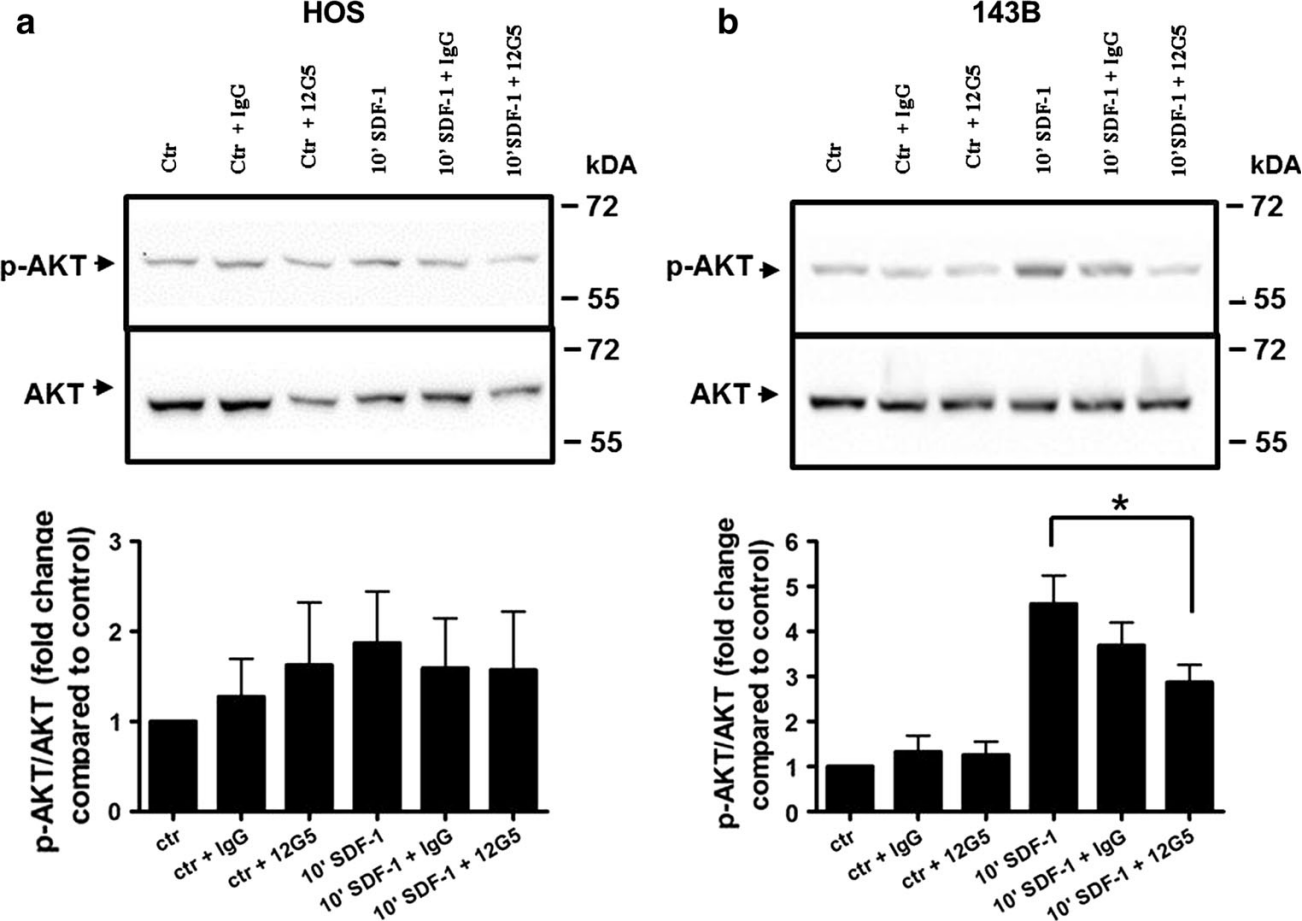
AKT signaling inhibition by the blocking anti- CXCR4 antibody 12G5 in 143B and HOS cells. Shown are p-AKT and total AKT levels in HOS (**a**) and 143B (**b**) cells that were left untreated (ctr) or stimulated 10 min with SDF-1 (10′ SDF-1) and were pre-incubated with IgG (ctr + IgG and 10′ SDF-1 + IgG) or 12G5 (ctr + 12G5 and 10′ SDF-1 + 12G5) antibodies. *Data* indicate mean ± SEM (*n* *=* 3) levels of p-AKT normalized to total AKT (*p ≤ 0.05 compared to control 10′ SDF-1 stimulation)

This pattern of CXCR4 expression in HOS and 143B cells was confirmed by the signaling response of the cells to CXCL12. Time-dependent transient phosphorylation of AKT and ERK upon stimulation with 100 ng/ml CXCL12 was only observed in 143B cells (Fig. 2c). Maximal stimulation was observed after 10 min of incubation with CXCL12 and amounted to a 22- and 2-fold increase of phospho-AKT and phospho-ERK, respectively, over basal levels when normalized to total AKT and ERK (Fig. 2d). HOS cells did not show any significant increase in phosphorylation of AKT and ERK when compared to the control cells after normalization to total AKT and ERK (Fig. 2a, b). CXCR4 dependent AKT signaling could by efficiently blocked in 143B cells by 12G5 while HOS did not show any stimulation and consequently any inhibition upon pre-incubation with 12G5 (Fig. 3a, b).

### CXCL12-provoked chemotaxis of HOS and 143B cells

**Fig. 4:**
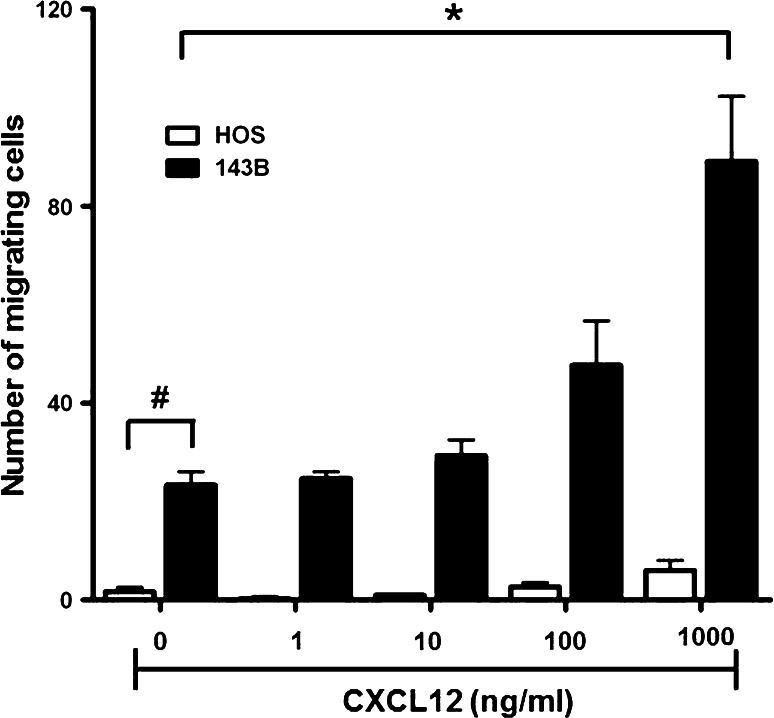
CXCL12 dose-dependent CXCR4-mediated chemotaxis of HOS and 143B cells. Cells were seeded in 48-well Boyden chambers on membranes of 8 μm pore size and chemotaxis to indicated concentrations of CXCL12 added to the medium in the bottom chamber was allowed for 4 h. Non-migrating cells adhering to the upper surface of the membrane were removed and cells that had migrated across the membrane and adhered to the bottom surface were fixed, stained with Diff Quick^®^ and counted under the microscope. *p *≤* 0.05 compared to 143B cells incubated in the absence of CXCL12; ^#^ *p* *≤* 0.05 compared to HOS cells in the absence of CXCL12

Migratory activity of the cell lines, considered as an in vitro indicator of in vivo metastatic activity, was assessed in a Boyden chamber migration assay in the absence and presence of CXCL12 in the lower chamber. The basal motility, indicated by the number of cells migrating across the filter in the absence of CXCL12, was 15-times higher in 143B than in HOS cells (Fig. 4). A maximal 3.7-fold increase in the number of migrating 143B cells in response to 1 μg/mL CXCL12 in the lower compartment of the Boyden chamber reflected the expected chemotactic response. HOS cells with only hardly detectable CXCR4 expression, on the other hand, showed only a minimal chemotactic response to CXCL12.

Altogether, the findings were consistent with the reported high metastatic activity of 143B compared to HOS cells and suggested that the CXCL12-stimulated CXCR4 signaling and migration activity of 143B cells contributed to their in vivo metastatic potential. Thus, the mAb 12G5 to human CXCR4, reported to inhibit the binding of human CXCL12 and downstream signaling of human CXCR4, was used to investigate the CXCR4 blocking principle in the 143B-*lacZ* cell-derived intratibial, spontaneously metastasizing OS mouse model, mimicking the human condition.

### Repetitive systemic treatment of SCID mice suffering from intratibial 143B-*lacZ* OS xenografts with CXCR4-blocking antibodies diminishes osteolysis and inhibits lung micro-metastasis

**Fig. 5:**
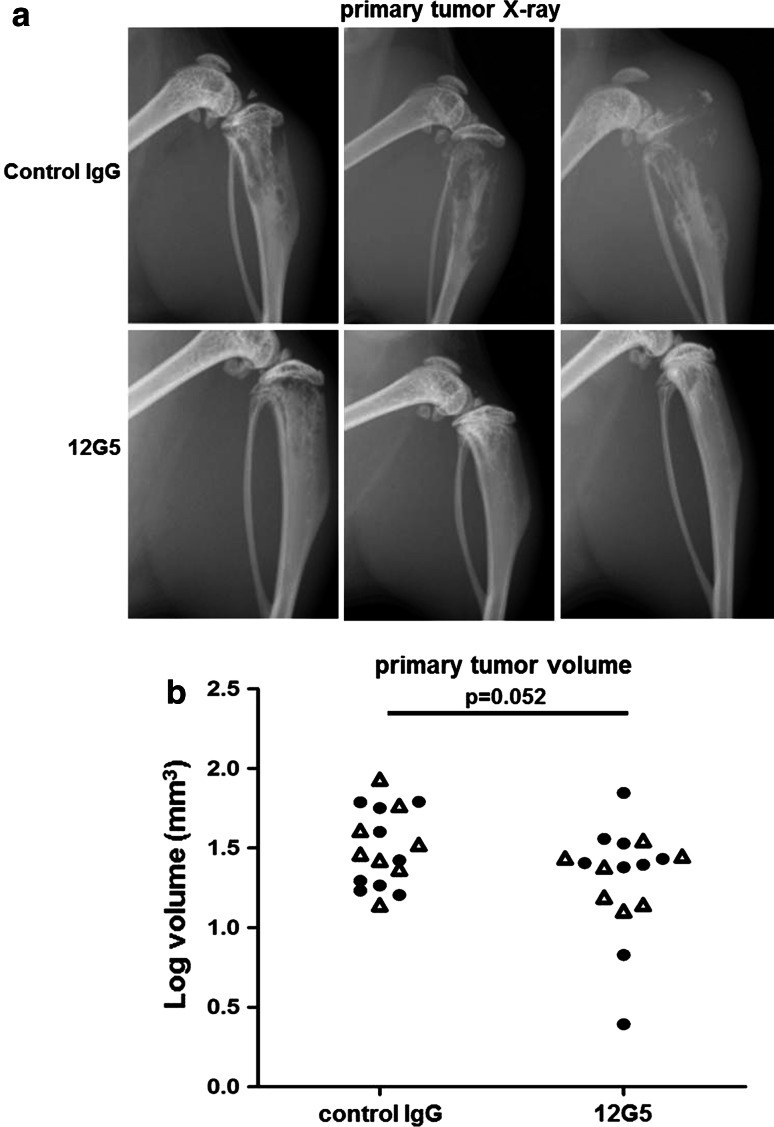
Effects of repetitive systemic administration of 12G5 CXCR4-blocking antibodies on intratibial primary tumor development of 143B-*lacZ* osteosarcoma cell xenografts. **a** Representative X-ray images of 143B-*lacZ* cell-derived tumors in severe combined immunodeficient mice treated with control mouse IgG (*top panels*) or with 12G5 CXCR4 antibodies (*bottom panels*). **b** Primary tumor volumes calculated from caliper measurements of tumor bearing tibiae in control mouse IgG- or 12G5 antibody-treated mice. The results are from two independent experiments with 7 (first experiment) and 6 (second treatment) (*open triangle*) and 9 (*filled circle*) mice per indicated group

**Fig. 6:**
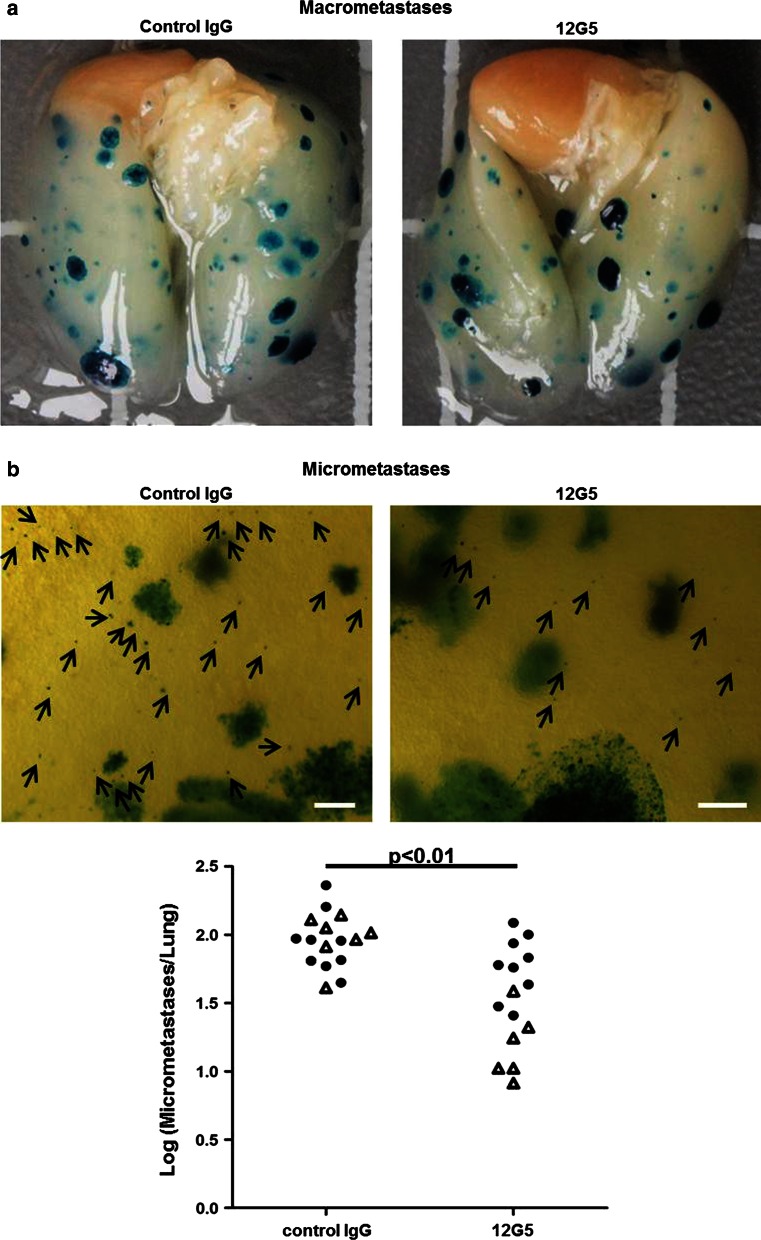
Micrometastasis-inhibiting effects of repetitive administration of CXCR4-blocking 12G5 antibodies in mice with intratibial 143B-*lacZ* cell-derived tumors. **a** Representative images of whole mounts of lungs of mice treated with mouse IgG (*left*) or with 12G5 CXCR4 antibodies (*right*). X-gal stained metastases appear in blue. **b** Microscopic images of representative areas of lung whole mounts (4-fold magnification, size bar = 10 μm) of 143B-*lacZ* cell tumor-bearing mice treated with mouse IgG (*left*) or with 12G5 CXCR4 antibodies (*right*). *Arrows* point to X-gal-stained micrometastases in *blue*. The total numbers of micrometastases per lung of indicated mice treated with mouse IgG or with 12G5 antibodies are shown in the *bottom panel*. The results were obtained in two independent experiments with 7 (*open triangle*) and 9 (*filled circle*) mice per indicated group. *p* < 0.01 was considered as statistically significant

Osteolysis in the proximal tibia, visualized by X-ray before sacrifice of the mice on day 20 after 143B-*lacZ* cell injection, was considerably less pronounced in animals treated in 4-day intervals by tail–vein injections of 200 μg mAb 12G5 compared to mice receiving the same doses of control mouse IgG (Fig. 5a). Mice treated with mAb 12G5 showed also a tendency (*p* = 0.052) to tumors with a smaller volume than those of control mouse IgG-treated animals (Fig. 5b). Thus, repetitive tail-vein bolus injection of CXCR4 antibodies inhibited the development of OS in the tibia from 143B-*lacZ* xenografts during the experimental period of 20 days. The ex vivo analysis of the X-gal stained metastatic lesions on lung surfaces showed that the repetitive treatment of the mice with mAb 12G5 compared to the treatment with control IgG had no effect on the number of outgrown lung macrometastases (>0.1 mm in diameter) at sacrifice on day 20 after intratibial tumor cell injection (Fig. 6a). However, mice treated with mAb 12G5 had significantly (*p* *<* 0.01) lower numbers of apparently minimally growing micro-metastases (<0.1 mm in diameter) than mice treated with mouse IgG (Fig. 6b), indicating that circulating CXCR4-blocking antibodies effectively inhibited the homing to the lung of 143B-*lacZ* cells disseminating from primary intratibial tumors.

## Discussion

Metastasis predominantly to the lung remains the leading cause of death in OS patients despite the implementation of neoadjuvant/adjuvant chemotherapy in the late 1970s [7]. Consequently, new treatment modalities targeting the complex metastatic process are needed. The chemokine CXCL12 has been recognized as an important chemoattractant of CXCR4 expressing metastasizing cells of numerous tumor types. CXCL12 has also been proposed as molecular cue guiding metastasizing OS cells to the lung where CXCL12 is expressed [12, 21, 35, 36]. Studies in OS mouse models of experimental metastasis, revealed by intravenous injections of tumor cells, indeed demonstrated the relevance of the CXCL12/CXCR4 axis in OS metastasis [24–26]. These were seminal and valuable proof of principle studies despite the fact that they employed mouse models of experimental metastasis, which rather incompletely reproduce the complex cascade of biological processes that are crucial in OS metastasis. In the present study, we therefore reinvestigated the CXCR4-blocking principle for OS treatment in the human 143B OS cell line-derived intratibial xenograft model in SCID mice that closely mimics the human disease with metastasis from the primary bone tumor to the lung.

The results presented here demonstrate that targeting the CXCL12/CXCR4 axis in the orthotopic mouse model of spontaneously metastasizing OS by repetitive tail-vein bolus injections of the anti-CXCR4 mAb 12G5 slowed down the osteolytic tumor development and, even more importantly in the context of OS, significantly reduced the number of primary tumor cells that disseminated to the lung where they were recognized as apparently non-growing micrometastases. This is an important finding in view of urgently needed novel strategies for more effective treatment of OS patients with metastatic disease. Bruland and co-workers could further demonstrate that the number of circulating micro-metastasis positively correlated with OS patient outcome [37] underlining the importance of finding efficient treatment modalities against these circulating cells as shown in the present study. However, the applied antibody treatment did not significantly affect the number of outgrowing lung macro-metastases. These observations likely reflect some limitations of the here used experimental OS mouse model, which is dependent on intratibial injection of the highly aggressive metastatic 143B OS cells. Despite taking any possible measures to avoid the escape of tumor cells from the injection site, we believe that it is almost inevitable that a small number of injected cells end up in the circulation and finally grow as metastases in the lung before effective onset of the antibody therapy. In an attempt to minimize the consequences of the above mentioned cell leakage and to further estimate the full potential of the herein presented antibody therapy strategy an upfront study administrating antibody prior to it cell injection is foreseeable. The evident disadvantage of such a treatment modality would be its lack of reflection of the patient situation. Moreover, in interpreting the data, it also has to be considered that approximately 25 % of 143B cells analyzed by FACS before injection showed at least temporarily non-detectable CXCR4 expression at the cell surface, and it is conceivable that such cells were inefficiently targeted by CXCR4 antibodies. Nevertheless, the number of lung micro-metastases was significantly reduced by antibody treatment, indicating that the CXCR4-blocking antibody 12G5 effectively interfered with the homing of metastasizing 143B cells to the lung, most likely through binding to the cells in the circulation. In addition we could show that the anti-CXCR4 antibody 12G5 efficiently blocked CXCL12 dependent AKT signaling, involved in migration and cell survival, essential for metastatic spread, in the highly metastatic human OS cells 143B, but not in their parental non-metastatic HOS cells which lack CXCR4 and are therefore unresponsive towards CXCL12. Interestingly, the metastasis inhibiting potential of mAb12G5 treatment observed in the present study was comparable to that reported for a tumor challenging experiment performed by Bertolini and co-workers [38]. In this report human Non-Hodgkin’s Lymphoma cells, pre-treated with mAb 12G5, were tail- vein injected into immunocompromised NOD/SCID mice and animals having received antibody-treated cells showed a significantly longer survival compared to control animals. In the same study diminished primary tumor development was observed at a moderate level, which is in agreement with the findings of the present study. Thus, all these observations taken together suggest, that, in the present study, tail-vein administered CXCR4 antibodies most effectively interfered with CXCL12-guided metastasizing, CXCR4 expressing 143B cells in the circulation. Furthermore we cannot exclude that also other mechanisms like 12G5 mediated ADCC (antibody dependent cellular cytotoxicity) via monocyte/macrophages or NK cells lead to cancer cell killing in the bloodstream. There the cells were likely exposed to the temporarily highest concentrations of the antibodies on their way to the lungs. In the developing primary bone tumor and in growing metastases in the lung, on the other hand, the antibodies likely had poor access to the tumor cells due to slow and inefficient delivery mechanisms in these tissues for proteins of that size. Consequently, the growth of tumor cells in the bone and in lung metastases was much less effectively inhibited by CXCR4 antibodies than CXCL12-guided and CXCR4-mediated chemotaxis of metastasizing tumor cells in the circulation.

In conclusion, the results of the present study demonstrate that a metastasis-suppressive CXCR4-blocking strategy is effective in an intratibial mouse model of spontaneously metastasizing, CXCR4-expressing OS. Consequently, the development of high affinity CXCR4-blocking agents with a long half-life and in formulations suitable for selective targeting of metastasizing OS cells in the primary tumor, the circulation and secondary organs appears to be a worthwhile approach to increase the efficacy of the treatment of metastasizing OS in combination with current treatment modalities or novel drugs.

## Abbreviations

OS: Osteosarcoma
GAPDH: Glyceraldehyde-3-phosphate dehydrogenase
FACS: Fluorescence-activated cell sorting
BSA: Bovine serum albumin
